# The ultrasonographic spectrum of toe dactylitis in psoriatic arthritis: a descriptive analysis

**DOI:** 10.1007/s10067-025-07395-y

**Published:** 2025-03-20

**Authors:** Garifallia Sakellariou, Nicolò Girolimetto, Ilaria Tinazzi, Marco Canzoni, Georgios Filippou, Alberto Batticciotto, Niccolò Possemato, Pierluigi Macchioni, Orazio De Lucia, Christian Dejaco, Luca Idolazzi, Carmelo Pirri, Annamaria Iagnocco

**Affiliations:** 1 Department of Internal Medicine and Therapeutics, Università Di Pavia, Pavia, Italy; 2 Istituti Clinici Scientifici Maugeri IRCCS Pavia, Pavia, Italy; 3 UO Interaziendale Medicina Interna Ad Indirizzo Reumatologico AUSL BO-IRCCS AOUBO, Bologna, Italy; 4 Unit of Rheumatology, ‘Sacro Cuore’ Hospital, Negrar, Italy; 5 ASL Rome 1 UOSD Reumatologia, Ospedale Nuovo Regina Margherita, 00153 Rome, Italy; 6 Department of Rheumatology, IRCCS Galeazzi-Sant’Ambrogio Hospital, Milan, Italy; 7 Department of Biomedical and Clinical Sciences, University of Milan, Milan, Italy; 8 Rheumatology Unit, Internal Medicine Department, ASST Sette Laghi, Ospedale Di Circolo, Fondazione Macchi, Varese, Italy; 9 Rheumatology Unit, Azienda Unità Sanitaria Locale-IRCCS Di Reggio Emilia, Reggio Emilia, Italy; 10 UOC Clinica Reumatologica, ASST Centro Traumatologico Ortopedico Gaetano Pini-CTO, Milan, Italy; 11 Department of Rheumatology, Hospital of Bruneck (ASAA-SABES), Teaching Hospital of the Paracelsius Medical University, Bruneck, Italy; 12 Department of Rheumatology and Immunology, Medical University Graz, Graz, Austria; 13 Rheumatology Section, Department of Medicine, University of Verona, Verona, Italy; 14 Department of Neurosciences, Institute of Human Anatomy, University of Padova, Padua, Italy; 15 Academic Rheumatology Center-DSCB Università Degli Studi Di Torino, AO Mauriziano Di Torino, Turin, Italy

**Keywords:** Dactylitis, Disability, Foot, Imaging, Psoriatic arthritis, Ultrasonography

## Abstract

**Introduction:**

Dactylitis is a hallmark of psoriatic arthritis (PsA). While its assessment is clinical, recently musculoskeletal ultrasonography (MSUS) has been applied to its monitoring. However, the evidence on MSUS application for toe dactylitis is limited. The aim of this study is to characterize the ultrasonographic features of toe dactylitis in PsA.

**Method:**

Patients with PsA and painful toe dactylitis were retrospectively identified from clinical records. Demographic and clinical variables were analyzed. Ultrasound images of the affected toe, allowing the assessment of grey scale (GS) and power Doppler (PD) were collected, to evaluate tenosynovitis, soft tissue oedema (STO), synovitis of metatarsophalangeal (MTP), proximal and distal interphalangeal (PIP, DIP) joints (all graded 0–3), and peritendonitis (PTI) at the MTP and PIP (graded 0–1). Clinical and ultrasonographic features were analyzed through descriptive statistics.

**Results:**

The study included 26 patients (30 toes) of which 9 (34.5%) females, with mean (sd) age of 46.8 (11.73). All but one patient had an oligoarticular phenotype. Tenosynovitis was the most frequent lesion, with GS abnormalities in 27/30 toes (90%) and PD in 25/30 (83.3%). STO was common (GS in 28/30 (93.33%) toes and PD in 20/30 (66.66%)). Synovitis was less common (63.33%, 46.66% and 33.33% of MTPs, PIPs and DIPs, respectively), while PTI was uncommon, with no patient presenting with PD.

**Conclusions:**

Ultrasound showed different elementary lesions in toe dactylitis confirming the complexity of this manifestation also at foot. These findings represent a first step toward the development of further imaging studies assessing toe dactylitis in PsA.

**Table:** 

**Key Points** • *Tenosynovitis and soft tissue oedema were the most common ultrasonographic elementary lesion in acute toe dactylitis in psoriatic arthritis.* • *Synovitis was less frequent and peritendonitis was very uncommon.* • *Musculoskeletal ultrasound confirms the presence of multiple lesions in painful toe dactylitis, confirming the complexity of this manifestation.*

## Introduction

Dactylitis, the uniform swelling of a hand or foot finger related to the simultaneous inflammatory involvement of all the articular and periarticular structures, is a clinical feature of spondyloenthesoarthritis. This manifestation is particularly peculiar to psoriatic arthritis (PsA), and it is of such relevance to be included in the classification criteria for the disease [1] and among the relevant domains to measure treatment efficacy [2]. The lifetime prevalence of dactylitis in patients with PsA can reach up to 70%, and in some cases can be an early manifestation, being the only sign of PsA for many years [3].

In patients with PsA, dactylitis might also have some prognostic implications, as it is significantly associated with higher disease activity, a lower probability of achieving minimal disease activity [4] and to a greater likelihood of radiographic damage [5].

The diagnosis of dactylitis is based on clinical means, although physical examination has a limited ability to discriminate between the different elementary lesions of this manifestation. Until recently, the instruments to assess dactylitis in clinical trials, such as the Leeds Dactylitis Index [6], were based on the clinical assessment, and this was reasonable as a limited number of trials with such outcome were conducted. However, in the last years the domain of dactylitis has been included in most clinical trials in PsA, and there have been several significant treatment innovations, involving both conventional synthetic disease modifying anti-rheumatic drugs (csDMARDs), in particular methotrexate [7], biologic DMARDs (bDMARDs) and targeted synthetic DMARDs (tsDMARDs) [8]. In this context, dactylitis outcomes are routinely introduced in clinical trials, and, recently, in a study with dactylitis as primary outcome has been performed [9]. All these novelties call for the necessity of more precise measures to follow this condition.

In the setting of inflammatory arthritis, including PsA, musculoskeletal ultrasonography (MSUS) has found a relevant field of application [10]. Unlike clinical assessment, MSUS is able to identify the elementary components of dactylitis, including tenosynovitis, synovitis, peritendonitis and, a peculiar feature of dactylitis, soft tissue oedema (STO) [11]. Ultrasound-detected abnormalities have recently been related to the diagnosis of PsA in patients with arthralgia [12], and subclinical alterations have been related to a more severe PsA phenotype [13].

Given the promising applications of MSUS in dactylitis, two scoring systems, developed with the aim to follow-up hand dactylitis in clinical studies, have been recently proposed. The DACTylitis glObal Sonographic (DACTOS) and the GLobal OMERACT Ultrasound DActylitis Score (GLOUDAS) scores were both created through a consensus process and tested in terms of reliability [14, 15]. DACTOS has also shown to be responsive after the administration of an effective treatment [16], however both instruments were not specifically designed for the assessment of dactylitis of the feet.

On the other hand, dactylitis at the feet is a common clinical scenario. In fact, this picture is more common at this level than in the hand, with the fourth finger being most frequently affected [17] and with an overall point prevalence of 17.5% [18]. In this area, the clinical detection and the assessment of dactylitis is even more challenging, as body weight and local trauma might act as confounders. To our knowledge, there is no available evidence on the frequency and distribution of dactylitis elementary MSUS lesions at the feet.

The purpose of the present study is therefore to describe the prevalence of the different elementary lesions in foot dactylitis identified through MSUS in a population of patients with PsA and active dactylitis.

## Methods

Patients with PsA, defined according to the Classification of Psoriatic Arthritis (CASPAR) criteria [1], consecutively seen in the rheumatology outpatients clinic of the Istituti Clinici Scientifici Maugeri, Pavia, Italy and presenting with dactylitis of the foot, were selected retrospectively from clinical records. Only patients with a recent onset of dactylitis, presenting with pain, were enrolled, since the focus of the study was to describe the ultrasonographic features of acute dactylitis. Additional inclusion criteria were an age ≥ 18 years and willingness to sign the informed consent form. Exclusion criteria were the following: recent (≤ 1 month) trauma in the involved area, recent (≤ 3 months) joint injections and concurrent diagnosis of other inflammatory arthropathies (e.g. gout). The study was approved by the Ethics Committee of the Istituti Clinici Scientifici Maugeri of Pavia (approval number 2731 CE).

The main demographic features (age and sex), clinical characteristics (clinical phenotype according to Moll and Wright [19], disease duration, duration of symptoms related to dactylitis, ongoing treatment), the intensity of pain measured by a Visual Analogue Scale (VAS) on a scale 0–10 and the Leeds Dactylitis Index (LDI) were recorded.

All patients had available musculoskeletal ultrasound (MSUS) assessment of the digits affected by dactylitis, with representative images depicting the entire finger and allowing the assessment of elementary ultrasonographic lesions of dactylitis. All MSUS examinations were performed by a single operator, with 14 years of experience of MSUS in rheumatology and involved in national and international studies, with high-end equipment (Esaote Mylab 70 or Samsung RS80 Evo machine, available at different times), equipped with high-frequency (6–18 MHz and 10–18 MHz, respectively) probes. Scans and machine settings were the same for all the patients included. Dorsal and plantar views of the finger were acquired, applying an adequate amount of gel to avoid pressure; the area was scanned in grey scale (GS) and power Doppler (PD) modalities. GS frequency was set in each patient to optimize resolution. For PD assessment, the color box was set to include the region of interest and the surrounding tissues, including the skin. The pulse repetition frequency (PRF) was set at 500 Hz, the color gain was set just below the level causing random noise artifacts to maximize sensitivity. Flexor and extensor tendons, the subcutaneous tissue, metatarsophalangeal (MTP), proximal (PIP) and distal (DIP) interphalangeal joints were evaluated in longitudinal and transverse view [20]. The elementary lesions previously described as components of dactylitis [14, 16] were evaluated, in particular flexor tenosynovitis, peritendonitis (PTI) at MTP and PIP, soft tissue oedema (STO) and synovitis at MTP, PIP and DIP. The elementary lesions were defined and evaluated according to the scoring system adopted for hand dactylitis in the DACTylitis glObal Sonographic (DACTOS) score [14, 16]. Briefly, flexor tenosynovitis was scored 0–3 for GS and PD in the most severely affected area, PTI was scored 0–1 for GS and PD, STO was scored 0–3 for GS and PD in the most severely affected area [11, 14], while the combined score for GS and PD, ranging from 0 to 3, proposed by EULAR and OMERACT, was adopted to score synovitis at the MTP, PIP, DIP [21].

Descriptive statistics, including mean and standard deviation (sd), median and interquartile range (IQR) and frequencies, were used to describe the demographic and clinical features of the population, as appropriate. Moreover, the frequency of each elementary lesion was described reporting frequencies and the median (IQR) score for each elementary lesion. A correlation analysis between VAS pain and the score of single ultrasound elementary lesions was carried using the Spearman correlation coefficient, with the patient as statistical unit, taking into consideration the most affected digit in case of multiple dactylitis; significance was set at 0.005 after applying the Bonferroni correction for multiple comparisons. Analyses were performed with MedCalc Statistical Software version 19.2.6 (MedCalc Software bv, Ostend, Belgium; https://www.medcalc.org; 2020).

## Results

A total of 26 patients were enrolled, presenting with a total of 30 foot digits affected by acute dactylitis, with 3 patients having multiple sites involved. The mean (sd) age was 46.8 (11.73), 9/26 (34.6%) patients were females. A single patient, presenting with the involvement of 3 digits, presented with a phenotype of symmetrical polyarthritis, while the remaining had asymmetrical oligoarthritis. The main demographic and clinical features of the population are summarized in Table 1.

**Table 1:** Demographic and clinical features of the population

Number of patients	26
Number of fingers with dactylitis	30
Females (n, %)	9 (34.6)
Age, years (mean, sd)	46.8 (11.73)
Disease duration, months (median, IQR)	24 (7.5–48)
Dactylitis duration, weeks (median, IQR)	4 (3–10.25)
Clinical phenotype (n, %)	
* Asymmetrical oligoarthritis*	25 (96.15)
* Symmetrical polyarthritis*	1 (3.85)
Treatment (n,%)	
* NSAIDS*	7 (26.9)
* systemic glucocorticoids*	2 (7.7)
* local glucocorticoids (injection)*	2 (7.7)
* methotrexate*	11 (42.3)
* sulphasalazine*	1 (3.85)
* leflunomide*	1 (3.85)
* adalimumab*	2 (7.7)
* secukinumab*	1 (3.85)
* no treatment*	2 (7.7)
*VAS pain (median, IQR)*	6 (4–7.75)
*LDI (median, IQR)*	1 (1–1)

*Sd;* standard deviation, *NSAIDs;* non-steroidal anti-inflammatory drugs, *VAS;* visual analogue scale, *LDI;* Leeds Dactylitis Index, *IQR;* interquartile range

**Fig. 1:**
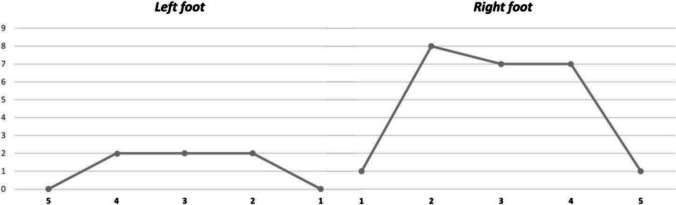
Frequency of dactylitis in each toe. Horizontal axis: affected toe; vertical axis: number of affected toes

The right foot was affected more frequently by dactylitis, with 24/30 (80%) cases presenting at this side, while the second finger was most frequently involved (10/29, 33.3%). The frequency of involvement of each finger is shown in Fig. 1.

**Fig. 2:**
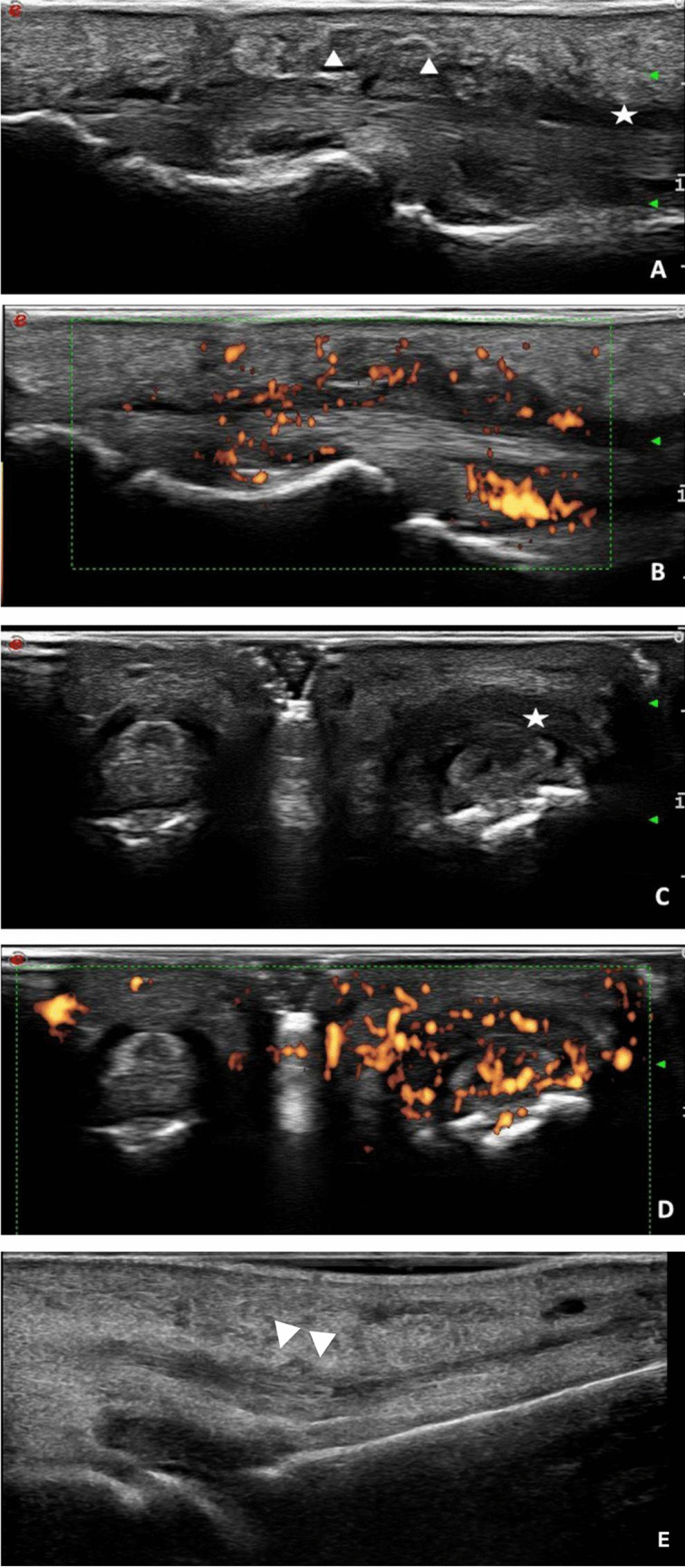
2.1 Longitudinal ultrasound views of toe dactylitis. **A**: Flexor tenosynovitis (white asterisk) with soft tissue oedema (white arrowheads). **B**: Presence of power Doppler signal (PDS) around the tendon fibers and in subdermal tissue. 2.2 Transverse ultrasound views of toe dactylitis. Comparison between a toe affected by dactylitis (right) and a healthy toe (left). **C:** The affected toe shows flexor tenosynovitis (white asterisk). **D**: In the affected toe there is PDS around the tendon fibers and minimally at the level of the subdermal tissue. 2.3. **E:** Longitudinal ultrasound view of toe dactylitis. Peritendinitis is shown by white arrowheads

Regarding the frequency of dactylitis elementary lesions, tenosynovitis and STO were the most common, with a frequency of 90% and 93.33%, respectively, while PTI was the least common, with an overall frequency at MTPS, and PIPs of 6.66%. No patient presented with peritendineous PD. An example of the US assessment is described in Fig. 2.

**Fig. 3:**
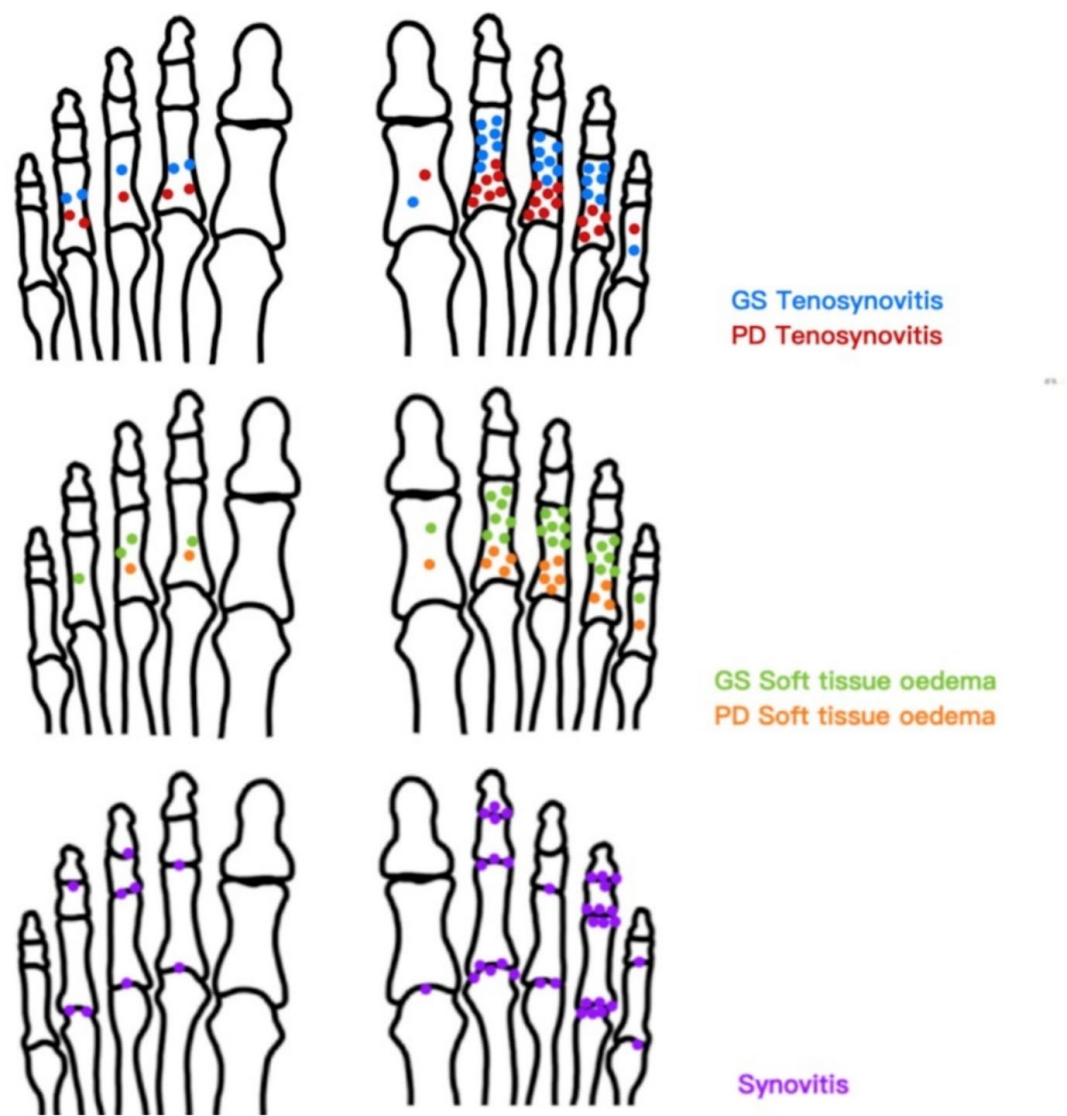
Distribution of dactylitis elementary lesions in each toe. GS: grey scale; PD: power Doppler

The frequencies of each elementary lesion are reported in Table 2, while the distribution depending on the affected finger is shown in Fig. 3.

**Table 2:** Median scores and prevalence of elementary ultrasound lesions

	Median (IQR) score	*N*, %
*Tenosynovitis GS*	2 (2–2)	27/30 (90)
*Tenosynovitis PD*	2 (1–2)	25/30 (83.3)
*STO GS*	2 (1–2.75)	28/30 (93.33)
*STO PD*	1 (0–1)	20/30 (66.66)
*Synovitis MTP*	1 (0–2)	19/30 (63.33)
*Synovitis PIP*	0.5 (0–1)	14/30 (46.66)
*Synovitis DIP*	0 (0–1)	10/30 (33.33)
*PTI GS MTP*	0 (0–0)	1/30 (3.33)
*PTI PD MTP*	0 (0–0)	0 (0)
*PTI GS PIP*	0 (0–0)	1/30 (3.33)
*PTI PD PIP*	0 (0–0)	0 (0)

*GS;* grey scale, *PD;* power Doppler, *MTP;* metatarsophalangeal, *PIP;* proximal interphalangeal joints, *DIP;* distal interphalangeal joints, *PTI;* peritendonitis, *STO;* soft tissue oedema

When assessing the correlation between VAS pain and elementary ultrasound lesions, the only variables with a significant correlation were GS STO and PD tenosynovitis, both showing a moderate correlation (Table 3).

**Table 3:** Correlation between VAS pain and elementary ultrasound lesions

	Correlation coefficient	p
*Tenosynovitis GS (0–3)*	0.465	p 0.0167
*Tenosynovitis PD (0–3)*	0.543	p 0.0041*
*STO GS (0–1)*	0.578	p 0.0020*
*STO PD (0–1)*	0.508	p 0.0081
*Synovitis MTP (0–3)*	0.222	p 0.286
*Synovitis PIP (0–3)*	−0.016	p 0.938
*Synovitis DIP (0–3)*	0.220	p 0.291
*PTI GS MTP (0–1)*	−0.241	p 0.279
*PTI GS PIP (0–1)*	0.000	p 1

*GS;* grey scale, *PD;* power Doppler, *MTP;* metatarsophalangeal, *PIP;* proximal interphalangeal joints, *DIP;* distal interphalangeal joints, *PTI;* peritendonitis, *STO;* soft tissue oedema

* significant results. Spearman’s correlation coefficient, significance level < 0.005 (Bonferroni correction for multiple comparisons)

## Discussion

The results of the present study represent the deepest insight on ultrasound in acute toe dactylitis available to date and represent a step towards the development of instruments allowing an accurate assessment for foot dactylitis. Despite the increasing relevance of an accurate assessment of all PsA domains in clinical research, the standard to assess the response of dactylitis to treatment still relies on clinical means, nonetheless the limitations of such approach and the impossibility to monitor single elementary lesions. The drawbacks of assessing dactylitis only clinically are even more evident when this manifestation occurs at the toes, which might be the only affected site in up to 66% of patients with dactylitis [16].

In this context, recent studies have evaluated the application of MSUS to patients with PsA and dactylitis affecting the hands [14, 15], with the aim to incorporate the many different elementary lesions of this condition into specific tools, developed for response monitoring in clinical research. However, so far these advancements have been limited to the setting of dactylitis of the hands, without any evidence for application at the level of the toes. In fact, the overall number of studies specifically focusing on toe dactylitis is limited, and this also applies to imaging studies, including ultrasonography and magnetic resonance imaging (MRI), with scarce information on the features of dactylitic involvement of the feet and the prevalence of elementary lesions. The existing gap in knowledge also reflects the difficulty in assessing the smaller structures of the toes, thus limiting the possibility of a precise MSUS assessment until recent years.

In the present study, we describe for the first time the ultrasonographic features of dactylitis with a specific focus on toe involvement, enrolling patients with PsA presenting with acute painful dactylitis. Although our sample size might seem small, it currently represents the largest population of patients with PsA and toe dactylitis characterized by clinical and ultrasonographic features. In fact, existing reports have mostly described the features of both finger and toe dactylitis, and the only studies assessing the MRI features of the toes enrolled a smaller number of subjects with spondyloenthesoarthritis [22, 23]. This also reflects the difficulty in assessing patients during the symptomatic and active phase of such manifestation. In fact, patients with dactylitis of the foot seldomly seek immediate medical care, as symptoms might be erroneously attributed to traumatism, and the self-limiting nature of the manifestation as well as the good response to NSAIDs tend to delay the referral to a rheumatologist.

Our population includes patients with relatively early disease, with a mean disease duration of 2 years, and with almost all patients presenting with the phenotype of asymmetrical oligoarthritis, confirming a higher frequency of dactylitis in this subgroup. The only patient presenting with polyarticular disease had indeed dactylitis of multiple toes. We found the highest prevalence of dactylitis at the right foot and in central toes. While we can hypothesize a relationship with the asymmetrical clinical presentation of arthritis, this information was not available in our population. At the MSUS examination, most patients presented with tenosynovitis and STO, in line with the typical findings described also at the level of the hands. These findings are also in keeping with the results of MRI studies on toe dactylitis, showing that flexor tenosynovitis is the most frequent elementary lesion, documented in all patients. While STO was also very commonly seen (90% of patients), the presence of synovitis, reported in 67% of patients, was not required to provide the typical sausage-like presentation. Moreover, inflammation surrounding the extensor tendons was also an infrequent finding (0–20% of patients) [22, 23]. The results of MRI studies however were not fully replicated by a subsequent ultrasonographic study, assessing both the fingers and the toes, where STO was more prevalent than tenosynovitis (74% compared to 52%) [22, 23].

The high prevalence of tendinous and soft tissue inflammation in the acute phases of dactylitis is in line with the report of the association of pain and tenderness with both GS and PD tenosynovitis and STO, detected by MSUS. On the other hand, the presence of synovitis had a negative association with symptoms and was detected more frequently in patients with non-painful dactylitis [24, 25], suggesting a greater relevance of this elementary lesion in the later phases of dactylitis. In keeping with this consideration, in our analysis we demonstrated a significant correlation between the entity of pain, assessed through the VAS scale, and STO abnormalities and tenosynovitis, despite the application of a very conservative correction. In our population a required inclusion criterion was represented by the presence of pain at the affected toe, thus determining the evaluation only of patients with acute dactylitis. In this setting, the higher prevalence of tenosynovitis over synovitis seems to be reasonable, as well as a high prevalence of soft tissue abnormalities, in line with previous descriptions of dactylitis of the fingers. In our population, in fact, the prevalence of elementary lesions reflects that reported on previous ultrasonographic studies assessing the hands [26].

Recently, Felbo et al. assessed patients with clinically identified dactylitis at the fingers and the toes, regardless of digit tenderness, by MSUS, using a 15 MHz transducer. In this population, including 55% of toe dactylitis, soft tissue thickening was the most frequent lesion (81%), while flexor tenosynovitis was present in 52% of patients. Compared to our study, synovitis was reported with a higher prevalence, with a frequency up to 68%[27]. The differences with our population are partly explainable by different inclusion criteria, as a different prevalence of elementary lesions depending on the phase of dactylitis has been described.

In our population the ultrasonographic finding of PTI surrounding the extensor tendon was particularly uncommon, with a lower frequency compared to what has been described at the fingers [16]. Moreover, no patient showed a positive PD signal at this level. While PTI is considered a specific finding of spondyloenthesoarthritis and specifically PsA, and reported in up to 65% of patients with PsA presenting with metacarpophalangeal swelling [28], this lesion seems to be of limited relevance in the context of dactylitis of the hands, and even less common at the feet. In the specific context of foot involvement, it should moreover be kept in mind that the assessment of periarticular abnormalities might be influenced by factors unrelated with PsA, such as soft tissue imbibition at the dorsal aspect of the feet related to venous insufficiency, determining a higher degree of uncertainty in defining PTI.

The results of our study confirm that the pattern of ultrasonographic involvement of toe dactylitis resembles that described at the hands. However, despite a similar distribution of elementary lesions, the immediate application of tools based on ultrasonography, developed on the hands, for the follow-up of toe dactylitis should be considered cautiously [14, 15]. In fact, the validity of these instruments should ideally be tested specifically also at the level of the feet, before considering their use in clinical research, as periarticular and peritendineous abnormalities at the feet might be influenced by many confounders, as described above. Moreover, the assessment of the small entheses of the fingers is gaining increasing relevance [15], however assessing these small structures at the toes can be even more challenging and suboptimal, also when high-end equipment is available. Based on our experience, the assessment of the small articular and periarticular structures at the toes should take place with high-frequency probes, with at least 18 MHz of frequency, also when evaluating larger structures, such as the joints and the tendons.

This study carries some limitations. As many patients are not seen in the acute phases of toe dactylitis, we were able to gather a limited sample size, also given enrollment at a single site. The retrospective design, based on existing images, and the cross-sectional data collection did not allow to evaluate the prognostic value of ultrasonographic findings. Moreover, the retrospective nature of the study determined the limited amount of clinical information that could be retrieved from the charts, and this, along with the small sample, did not allow to assess the association of ultrasonographic findings and clinical measures. There was no sufficient data to calculate composite measures of disease activity such as the Disease Activity Index for Psoriatic Arthritis (DAPSA). A further limitation is the fact that there was an exchange of ultrasound machines during the study period potentially impacting the sensitivity to detect elementary lesions. Despite these drawbacks, the present work has the merit to present for the first time a population of patients with PsA in which the ultrasonographic findings of toe dactylitis are specifically addressed and to describe the pattern of involvement.

While dactylitis is included among the domains that need to be assessed in clinical trials in patients with PsA, the assessment of toe involvement remains an uncovered area, with the need to develop and validate instruments to reliably assess and monitor such manifestation. Our results demonstrated a similar pattern of elementary lesions in acute toe dactylitis to that of finger dactylitis, with tenosynovitis and STO representing the most frequent lesions. Moreover, we demonstrated a significant correlation between pain and the presence of STO and tenosynovitis. Prospective multicenter study in a large cohort will enable subgroup analyses, correlation with clinical scores, reliability analysis and evaluation of prognostic impact. In addition, clinical trials could include ultrasound assessment of dactylitis as a possible outcome.

## Data Availability

The datasets generated during and/or analyzed during the current study are available from the corresponding author on reasonable request.
